# HNRNP G and HTRA2-BETA1 regulate estrogen receptor alpha expression with potential impact on endometrial cancer

**DOI:** 10.1186/s12885-015-1088-1

**Published:** 2015-02-27

**Authors:** Marc Hirschfeld, Yi Qin Ouyang, Markus Jaeger, Thalia Erbes, Marzenna Orlowska-Volk, Axel zur Hausen, Elmar Stickeler

**Affiliations:** 1 Department of Obstetrics and Gynecology, University Medical Center Freiburg, Hugstetterstr 55, 79106 Freiburg, Germany; 2 German Cancer Consortium (DKTK), Heidelberg, Germany; 3 German Cancer Research Center (DKFZ), Heidelberg, Germany; 4 Department of Obstetrics and Gynecology, Tongji Hospital of Tongji University, Shanghai, China; 5 Institute of Pathology, University Medical Center Freiburg, Freiburg, Germany; 6 Department of Pathology, Maastricht University Medical Center, Maastricht, The Netherlands

**Keywords:** HNRNPG, HTRA2-BETA1, Estrogen receptor alpha, Endometrial carcinoma, Prognostic significance, Alternative splicing

## Abstract

**Background:**

Estrogen receptor alpha (*ERa/ESR1*) expression is regulated by alternative splicing. Its most frequently detectable exon7 skipping isoform (*ERaD7*) is a dominant negative variant. Elevated expression of *ERaD7* was already detected in endometrial cancer (EC), while its potential prognostic significance has not been characterized so far. Exon7 contains potential binding sites for the two functional splicing regulatory opponents, HNRNPG and HTRA2-BETA1 known to trigger opposite effects on EC outcome.

This study served to elucidate the influence of HNRNPG and HTRA2-BETA1 on *ERa* exon7 splicing regulation and the impact of *ERaD7* concentration on type 1 EC outcome.

**Methods:**

Functional *in vitro* experiments for HNRNPG and HTRA2-BETA1 in regard to the regulatory impact on endogenous and exogenous *ERaD7* splicing were performed. Additionally, real-time PCR determined mRNA levels of *ERaD7*, *HNRNPG* and *HTRA2-BETA1* in 116 type 1 EC patients.

**Results:**

HNRNPG and HTRA2-BETA1 were found to be specific regulators of *ERa* exon7 splicing. While HTRA2-BETA1 promoted exon7 inclusion, HNRNPG antagonized this effect by inducing exon7 skipping (p = 0.004). *ERaD7* was detected in 71 out of 116 type 1 EC specimens. Statistical analyses revealed an inverse correlation between *ERaD7* mRNA levels and tumor grading (p = 0.029), FIGO stage (p = 0.033) as well as lymph node metastases (p = 0.032), respectively. Furthermore, higher *ERaD7* expression could be correlated to an improved disease-specific survival (p = 0.034).

**Conclusions:**

Our study demonstrates antagonistic regulatory effects of HNRNPG and HTRA2-BETA1 on *ERa* exon7 splicing with potential impact on type 1 EC clinical outcome due to the consecutively variable expression levels of the *ERa* isoform *D7*.

**Electronic supplementary material:**

The online version of this article (doi:10.1186/s12885-015-1088-1) contains supplementary material, which is available to authorized users.

## Background

**Figure 1:**
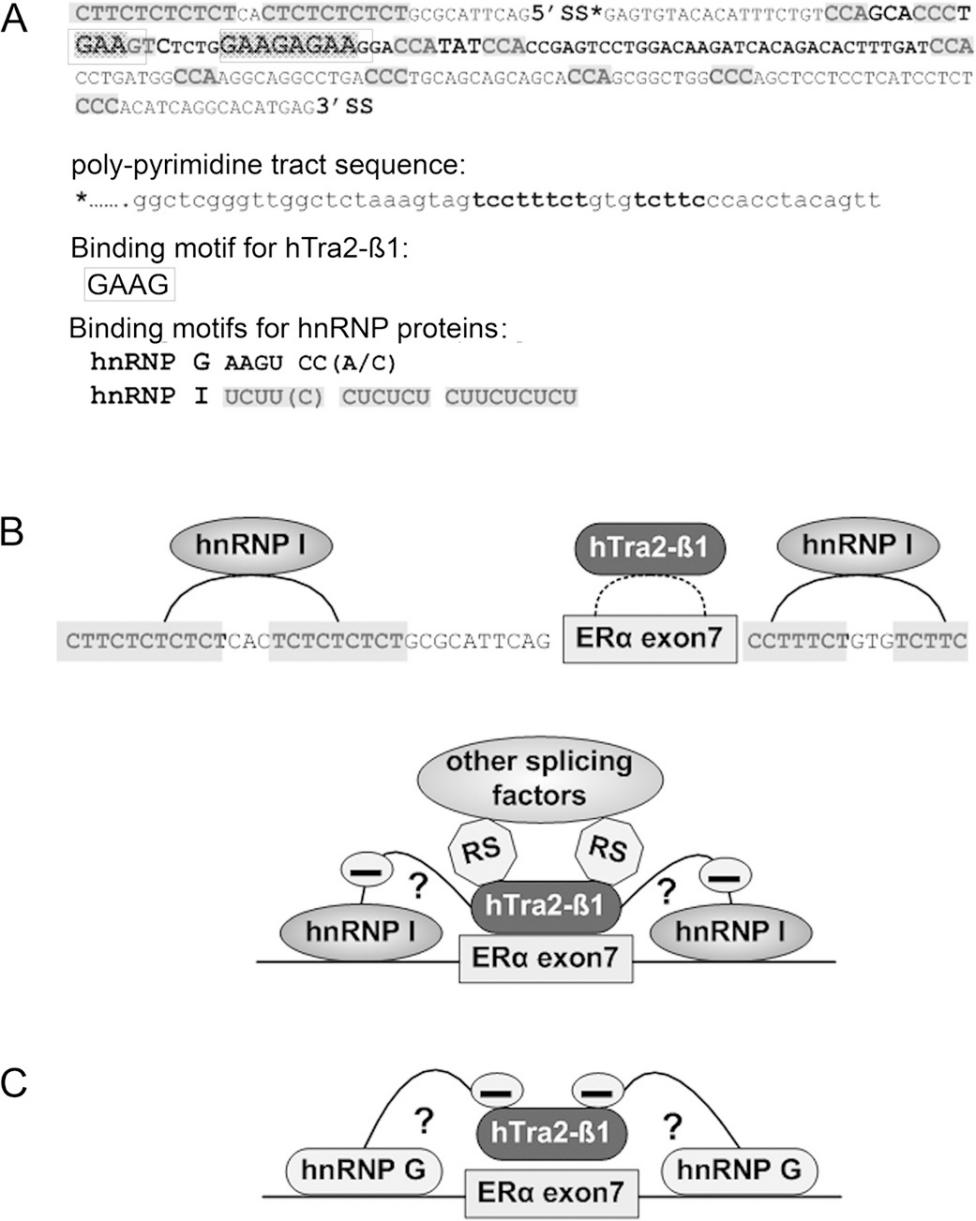
**Sequence analyses of*ERa*exon7 and potential mode of action of HNRNPG and HTRA2-BETA1 on*ERa*exon7 splicing regulation. (A)** Sequence analyses of *ERa* exon7. SS*: splice site; RS: arginine/serine rich domain of HTRA2-BETA1 (domain is required for protein-protein interaction and recruiting of other splicing factors to exons). *ERa* exon7 sequence is shown between 5’SS and 3’SS. Its poly-pyrimidine tract sequence is shown upstream of 5’SS and its 3’ intron sequence is shown downstream of 3’SS. Binding motifs of each splicing factor are stated out below exon7 sequence and are indicated in original sequence, respectively. **(B)** The antagonizing effect of HTRA2-BETA1 to HNRNP I is shown, the mechanism of this function is not clear. **(C)** The antagonizing effect of HNRNP G on HTRA2-BETA1 is shown. We propose that these two factors antagonize each other in RNA binding in a concentration dependent manner.

Endometrial cancer (EC) is the most common gynecological malignancy in the western world and accounts for 6% of all cancers in females [1]. The incidence is estimated at 15–20 per 100,000 women per year and it mainly affects peri- and postmenopausal women, with 89% of cases occurring between 65–69 years of age [2,3]. EC is classified into two subtypes: the estrogen-dependent type 1 with a background of excessive exposure to estrogen unopposed by progesterone and the estrogen-independent type 2 [4]. The lack of expression of estrogen receptor alpha (*ERa/ESR1*) in type 1 EC was found to be associated with poor differentiation of cancer tissues and poor survival rates of EC patients, respectively [5,6], supporting the hypothesis of a direct involvement of ERa in EC tumorigenesis and progression. The expression of ERa in normal or malignant endometrial tissue is subjected to alternative splicing modulating its biological function [7]. Several ERa splice variants with varying functional differences were described. ERa isoform skipping exon4 (ERaD4) misses the ability to bind to DNA or ligands, thus cannot stimulate estrogen-dependent gene expression. ER variants skipping exon3 (ERaD3) or exon7 (ERaD7) are referred to as dominant negative, since they interfere with normal ERa function, but cannot activate regular ERa-mediated transcription [8]. ERaD5, a constitutive mutant variant, is characterized by the capability to activate transcription of ER-dependent genes without binding to a ligand [8,9]. ERaD4, D5 and D7 were found in EC and physiological endometrium [8]. The *ERa* exon7 skipping (*ERaD7*) isoform has been identified as the most common phenotype in EC and breast cancer and encodes for a protein lacking a portion of the hormone binding domain [7,10]. This isoform represents a dominant negative variant for both ERa and ER beta [7,10]. Induced *ERaD7* expression has been detected in the proliferative compared to the secretory phase of endometrial tissue [11] and also in well to moderately differentiated EC in comparison to poorly differentiated EC [12]. Besides these findings and an influence on estrogen therapy sensitivity in schizophrenic patients [13], the clinical significance of *ERaD7* in estrogen related cancer has not been elucidated yet. Particularly the regulation of *ERa* mRNA processing is not well understood, despite ERa exon 7 contains potential binding sites for the two antagonistic splicing factors HTRA2-BETA1 and HNRNPG (Figure 1). Recently our group was able to link alternative splicing regulation to EC tumor biology and clinical outcome [14] and identified HNRNPG and HTRA2-BETA1 as independent prognosticators for EC type I progression-free survival. Their antagonizing effects on alternative splicing processes were directly reflected by their opposite effects on EC biology.

Since alternative splicing is a pertinent control mechanism of gene expression with consecutive impact on cellular processes like growth, apoptosis, invasion and metastasis, respectively [15], we intended to elucidate the potential regulatory influence of HNRNPG and HTRA2-BETA1 on *ERaD7* isoform expression profile in type 1 EC, as well as its potential impact on clinico-pathological characteristics and clinical outcome.

## Methods

### Patients and tissue samples

One hundred and sixteen consecutive patients with type 1 EC, who were treated at the Gynecological Hospital of University Medical Center Freiburg between November 1997 and December 2005, were included in this study. Median age of patients at the time of diagnosis was 65. Patients receiving hormone replacement therapy prior to surgery were excluded from the study. All patients underwent hysterectomy, salpingo-oophorectomy and pelvic lymphadenectomy (according to the current national guidelines), and were properly staged according to the International Federation of Obstetrics and Gynecology (FIGO) classification at the time. Tissue samples were obtained at the time of surgery and collected in the tumor tissue bank of Comprehensive Cancer Center Freiburg (CCCF), Germany. The institutional review board of CCCF and the local ethical committee of the University Medical Center Freiburg approved and licensed the investigation protocol of this study (#32409). All patients involved gave their informed consent prior to inclusion in this study.

Paraffin embedded tissue specimen from hysterectomies were obtained from the Institute of Pathology in University Medical Center Freiburg. All haematoxylin-eosin stained slides were reviewed by specially trained pathologists (AzH, MOV). Histological classification was performed according to the World Health Organization 2003 system [16] into well differentiated (G1; n = 33), moderately differentiated (G2; n = 59), and poorly differentiated (G3; n = 24), respectively. Most patients neither had regional lymph node metastases (81.9%) nor distant organ metastases (68.1%). Cancer relapse was found in 17 patients during follow up (14.7%). The time to relapse ranged from 10–101 months after surgery. During follow up nine patients with recurrence died from EC and one from other cause. Seven recurrent patients were under further follow up for an additional median time of 17 months (range 0.3-42 months, Table 1).

**Table 1:** **Clinico-pathological features of patient cohort**

	Type I EC
	(n = 116)
**Age (years)**	
<65	
≥65	
**Histoligical type**	
Enodometrioid adenocarcinoma	102 (87.9%)
Adenosquamouse carcinoma	14 (12.1%)
**WHO Grade**	
G1	34 (29.3%)
G2	59 (50.9%)
G3	23 (19.8%)
**Tumor size**	
1	86 (74.1%)
2	14 (12.1%)
3	14 (12.1%)
4	2 (1.7%)
**LN status**	
Negative	95 (81.9%)
Positive	14 (12.1%)
Unknown	7 (6.0%)
**Metastases**	
Negative	79 (68.1%)
Positive	7 (6.0%)
Unknown	30 (25.9%)
**Lymphagiosis**	
Negative	24 (20.7%)
Positive	26 (22.4%)
Unknown	66 (56.9%)
**FIGO stage**	
I	51 (44.0%)
II	7 (6.0%)
III	24 (20.7%)
IV	10 (8.6%)
Unknown	24 (20.7%)
**Postoperative therapy**	
No therapy	35 (30.2%)
Brachytherapy	41 (35.3%)
Radiotherapy	28 (24.2%)
Chemotherapy	2 (1.7%)
Chemotherapy & radiotherapy	6 (5.2%)
Unknown	4 (3.4%)
**Recurrent EC and outcome**	
EC recurrent	17 (14.7%)
EC related death	9 (7.8%)
Other related death	1 (0.9%)
Further-on follow up	7 (6.0%)

### RNA extraction from paraffin embedded tissue and cDNA synthesis

Each paraffin block used for RNA extraction was histologically assessed with regard to tumor homogeneity to guarantee a tumor cell content of more than 90%. Total tissue RNA was extracted by using the High Pure RNA Paraffin Kit (Roche, Mannheim, Germany) according to the manufacturer’s protocol. RNA quality was controlled by densitometry and accepted with A260/280 > 1.7. RNA integrity was controlled on a 2100 Accessories & Spare Parts system (Agilent Technologies, Waldbronn, Germany). Prior to RT-PCR, each RNA sample was digested with 2.0 U DNase I (Roche, Mannheim, Germany) at 37°C for 45 min to eliminate genomic DNA (gDNA) contamination. Four μg of purified RNA were transcribed into cDNA using M-MLV reverse transcriptase (Promega, Mannheim, Germany) and 10 pM random hexamer primers (New England Biolabs GmbH, Frankfurt, Germany) in a total volume of 50 μl.

### Real-time quantitative PCR

Primers used for real time PCR were all designed in an exon flanking way, except for *ERa* standard primers, which were located in *ERa* exon1 (Additional file 1: Table S1). Since *ERa* exon1 is constitutively transcribed in all *ERa* mRNA isoforms, we used this amplicon to represent the total *ERa* transcript level. *ERaD7* sense primer was located in conjunction part of *ERa* exon6 and 8 and the antisense primer in *ERa* exon8. This primer pair was designed to exclusively detect the *ERaD7* isoform.

Samples of cDNA were heated to 95°C for 5 min followed by 45 cycles of 95°C 20 s, 60°C 20 s, 72°C 20 s. Expression of each gene was aggregated and then normalized against housekeeping gene (HKG) RPS18. Relative expression levels were calculated using the following formula: Ratio = E _target_^ΔCt target (control –target)^ / E _HKG_^ΔCt HKG (control - HKG)^ [17]. All PCR analyses were performed in triplicates, while arithmetic mean of data served as base for subsequent statistical analysis.

### Plasmid construction

Full length of *HNRNPG* cDNA (NCBI Reference Sequence: NM_001164803.1) was subcloned into the mammalian expression vector pCMV-Script (Stratagene, Agilent Technologies, Waldbronn, Germany). The pCMV-Script vector was digested by EcoRV restriction enzyme (Fermentas, St. Leon-Rot, Germany) at 37°C for 1 hour. After digestion, blunt vector ends were dephosphorylated by Antarctic phosphatase (New England Biolabs, Frankfurt, Germany). *HNRNPG* full length cDNA was phosphorylated by Polynucleotide Kinase (New England Biolabs, Frankfurt, Germany). Dephosphorylated vector and phosphorylated insert were purified with High Pure PCR product purification Kit (Roche, Mannheim, Germany) prior to ligation. Blunt end ligation was achieved by using T4 DNA ligase (Fermentas, St. Leon-Rot, Germany) at 4°C overnight. *HTRA2-BETA1* expression plasmid was kindly provided by Prof. Stefan Stamm (Stamm’s Lab at Department of Molecular & Cellular Biochemistry, University of Kentucky, Lexington, U.S.A.). *HNRNPG* and *HTRA2-BETA1* shRNA plasmids were obtained from Santa Cruz (Santa Cruz Biotechnology Inc., Santa Cruz, U.S.A.).

#### ERa exon7 minigene construction

**Figure 2:**
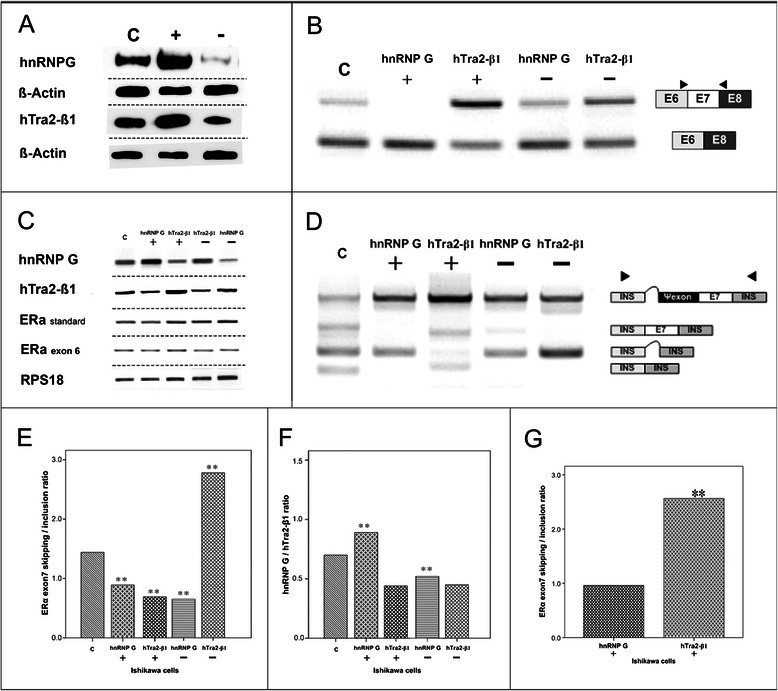
**Functional implications of HNRNP G and HTRA2-BETA1 in EC*in vitro.*(A)** HNRNP G and HTRA2-BETA1 protein expression in Ishikawa cells transiently transfected with expression and knock-down plasmids; (−) shRNA and (+) expression plasmid for HNRNPG and HTRA2-BETA1; **(C)** control: empty pCMV-plasmid. *HKG:Beta-Actin*. Western blot. **(B)** Influence of *HTRA2-BETA1* and *HNRNPG* mRNA-levels on endogenous *ERa-*exon7 mRNA splicing. **(C)** cells transfected with: control: empty pCMV-plasmid; **(HTRA2-BETA1+)** HTRA2-BETA1-expression-plasmid; **(HTRA2-BETA1 −)** HTRA2-BETA1-shRNA; **(HNRNPG +)** HNRNPG-expression-plasmid; **(HNRNPG −)** HNRNPG-shRNA. RT-PCR. **(C)***HNRNPG, HTRA2-BETA1, ERa*-standard and *ERa*-exon6 mRNA expression in differently treated Ishikawa cells. **(C)** control:pCMV-plasmid; **(HTRA2-BETA1 +)** HTRA2-BETA1-expression-plasmid; **(HTRA2-BETA1−)** HTRA2-BETA1-shRNA; **(HNRNPG +)** HNRNPG-expression-plasmid; **(HNRNPG −)** HNRNPG-shRNA. *HKG:RPS18*. RT-PCR. **(D)** Exogenous level of *ERa*-exon7 splicing pattern. Influence of overexpression (+) and knock-down (−) HNRNPG and HTRA2-BETA1 on alternative *ERa*-exon7 minigene expression. In untransfected control cells, the reporter gene was alternatively spliced into 4 isoforms, two precisely spliced isoforms are exon7-skipping (137bp) and exon7-inclusion (321bp). Two lariat containing isoforms are: one containing a part of intron sequence between INS-exon2 and -3 (210bp), another containing an additional pseudo-exon from exon7 5’ intron sequence (544bp, all four isoforms were verified by sequencing). RT-PCR. **(E,F)***ERa* exon7 alternative splicing regulation by HTRA2-BETA1 and HNRNPG in Ishikawa cells. **(E)** ERa-exon7 skipping/inclusion ratio; **(F)** HNRNPG/HTRA2-BETA1 ratio in differentially treated Ishikawa cells. **(C)** control:pCMV-plasmid; **(HTRA2-BETA1 +)** HTRA2-BETA1-expression-plasmid; **(HTRA2-BETA1 −)** HTRA2-BETA1 shRNA; **(HNRNPG +)** HNRNPG-expression-plasmid; **(HNRNPG −)** HNRNPG-shRNA. **(G)***ERa* exon7 skipping/inclusion mRNA ratio difference between HTRA2-BETA1overexpression and HNRNPG overexpression group. **(HTRA2-BETA1 +)** HTRA2-BETA1 overexpression; **(HNRNPG +)** HNRNPG overexpression; **ERa exon7 skipping/inclusion ratio between the two groups was statistically significant p= 0.004. *PCR-based tests originate on arithmetic mean of triplicate analyses. Student-T-test was applied for data shown in E-G, while statistical significance was assumed at p<0.05 at the two-sided test. Representative gel images in B-D demonstrate one out of three repeats.*

*ERa* exon7 together with part of its upstream and downstream intron sequence was subcloned into pCMV-INS plasmid. pCMV-INS plasmid contained insulin (*INS*) gene exon2 and exon3. Vector plasmid was digested between *INS* exon2 and exon3 with Pfl23II restriction enzyme (Fermentas, St. Leon-Rot, Germany). Vector dephosphorylation, insert phosphorylation and ligation were conducted as described above. The amount of insert for ligation was calculated according to following formula: Insert mass (ng) = 6× Insert length (bp) / Vector length (bp) × Vector mass (ng). Plasmids were subsequently verified by sequence analyses (GATC BIOTECH, Konstanz, Germany). Plasmid-relevant PCR products are shown in Figure 2D.

### Cell culture and transfection

Human EC cell line Ishikawa and human cervical cancer cell line HeLa were maintained in GIBCO® DMEM media (Invitrogen, Karlsruhe, Germany) supplemented with 10% fetal bovine serum (Invitrogen, Karlsruhe, Germany). Cells (150 × 10^3^/well) were seeded in 6-well plates (Ø 30 mm) 24 hours before transfection, leading to a cell confluency of 60-70%. Plasmid DNA was transfected into cells via polyethylenimine (PEI) transfection. Empty pCMV-Script vector was used as a transfection control (Additional file 1: Table S2).

Endogenous *ERa* exon7 splicing pattern were detected in differently treated Ishikawa cells after single *HNRNPG* or *HTRA2-BETA1* plasmid transfection, while exogenous splicing pattern were analyzed in co-transfected HeLa cells with the *ERa* exon7 minigene as reporter.

### RNA and protein extraction

Total cellular RNA and protein were extracted 48 hours after transfection applying TRIzol® reagent (Invitrogen, Karlsruhe, Germany) isolation protocol. Before RT-PCR, DNase I digestion was used to exclude gDNA contamination as well.

### Western blot for hnRNP G and HTRA2-BETA1 detection

Western blot analyses were performed to evaluate the *HNRNPG* and *HTRA2-BETA1* expression plasmid as well as shRNA plasmid efficacy after transfection. The immune complexes were visualized by an ECL assay (Figure 2A). Rabbit polyclonal IgG HNRNPG (RBMX) antibody (sc-48796, Santa Cruz Biotechnology, Inc.) and rabbit polyclonal IgG HTRA2-BETA1 antibody [18] (provided by Prof. Stefan Stamm, (Stamm’s Lab, Department of Molecular & Cellular Biochemistry, University of Kentucky, Lexington, U.S.A.)) were used.

### ERa exon7 detection in transfected Ishikawa and HeLa cells

The primer design for *ERa* exon7 amplification (amplicon ranging from exon6 to exon8) allowed the detection of both exon7 inclusion and skipping isoforms. Since *ERa* exon6 is also subject to alternative splicing, *ERa* exon6 as well as *ERa* standard (primers located in exon1) PCR assays were used as *ERa* transcript level control for differently transfected cells (primer sequences: Additional file 1: Table S1). Conditions for *ERa* standard, exon 6 and exon7 PCR were as follows: 95°C 5 min, followed by 35 cycles 95°C 20 s, 60°C 20 s, 72°C 20 s.

The primers for the pCMV-INS based were complementary to *INS* exon 2 and exon 3, respectively and designed to detect different splicing pattern of *ERa* exon7 minigene after co-transfection. Post co-transfection PCR: 95°C 5 min, followed by 45 cycles 95°C 20 s, 60°C 15 s, 72°C 45 s. PCR products were separated by gel electrophoresis and quantitative analysis was conducted by application of imageJ software (http://rsbweb.nih.gov/ij/).

### Sequence analysis

PCR products of endogenous and exogenous *ERa* exon7 alternatively spliced isoforms were subcloned into the CloneJET™ PCR Cloning Kit (Fermentas, St. Leon-Rot, Germany). PCR products of the *HNRNPG* expression plasmid, *ERa* exon7 minigene and *ERa* exon7 isoform PCR products were sequenced by GATC BIOTECH (Konstanz, Germany). Furthermore, all PCR amplicons produced in different analyses were subject to verification via sequence analysis. Sequencing results were compared with NCBI reference sequences (http://www.ncbi.nlm.nih.gov/).

### Statistical analyses

The expression levels of *ERa* were categorized as follows (normalization against RNA of HKG *RPS18*) for consecutive quantification:

group 0: no *ERa* standard mRNA detectable; group 1: *ERa* standard mRNA amount ≤0.81; group 2: *ERa* standard mRNA amount >0.81, due to the mean mRNA level within *ERa* standard positive samples being 0.81. *ERaD7* mRNA levels were defined as group 0: no *ERaD7* mRNA detectable; group 1: *ERaD7* mRNA amount ≤0.80; group 2: *ERaD7* mRNA amount >0.80, due to the mean *ERaD7* mRNA level within *ERaD7* positive ones being 0.80.

The *D7* real time PCR results were analyzed in regards to potential correlations with clinicopathological data by Spearman’s correlation test. Univariate and multivariate analyses were performed with Kruskal-Wallis H test and general linear model, respectively. When performing survival analyses, the records of patients who died of EC were considered to be uncensored; the records of patients who were alive during follow up or who died from other diseases were considered to be censored. Univariate analyses of disease-specific survival and progression-free survival were performed with Kaplan-Meier life-table curves and compared using the Log rank test. Multivariate prognostic analyses used multivariate Cox regression test in a forward step wise manner [19]. Student T test was used for RT-PCR results analyses. Statistical significance was assumed at p ≤ 0.05 at the two-sided test (SPSS 15.0 software, SPSS Inc.).

## Results

### HTRA2-BETA1 and HNRNPG as antagonistic regulators of ERa exon7 splicing

Functional experiments in Ishikawa endometrial cancer cells with transient transfection of *HTRA2-BETA1* and *HNRNPG* expression plasmids revealed that endogenous exon7 inclusion was specifically induced by HTRA2-BETA1. In contrast, HNRNPG acted as a splicing inhibitor with induced levels of exon7 skipping (Figure 2B). As a consequence, the exon7 skipping/inclusion ratio was significantly higher in HNRNPG in comparison to HTRA2-BETA1 overexpression (Figure 2B, C, F, Additional file 1: Table S4). However, expression of endogenous ERa standard as well as ERa exon6 was not affected by the two splicing factors (Figure 2C).

Employing an *ERa* exon7 reporter gene the findings of a high specificity of the HTRA2-BETA1 and HNRNPG effects were confirmed on the *in vitro* level (Figure 2D).

### Induced ERaD7 expression is correlated to favorable clinico-pathological parameters

In type 1 EC, *ERa* mRNA expression could be detected in 87 samples (75%) of which 71 (61.2% of the complete cohort) expressed the *ERaD7* isoform.

On the basis of categorization in groups 0–2 (*see Methods, statistical analyses*) both *ERa* standard and *ERaD7* mRNA levels were found to be inversely correlated to grading (−0.317, p = 0.001) and FIGO stage (−0.222, p = 0.033). Furthermore, increased *ERaD7* mRNA levels were detected in tumors without regional lymph node metastases (correlation coefficient = −0.206, p = 0.032, Table 2). The observed differences in *ERaD7* mRNA levels between well to moderately and poorly differentiated cancers, FIGO stage I/II and III/IV, as well as lymph negative and positive groups were all statistically significant (p = 0.030, p = 0.034, p = 0.032, respectively, Kruskal-Wallis Test, Table 3).

**Table 2:** **Correlation of** ***ERa*** **standard and** ***ERaD7*** **expression with clinico-pathological features and** ***HTRA2-BETA1*** **(Spearman’s correlation test)**

Spearman’s		FIGO	Grade	T	LN	M	L	hTra2β1
		(I/II vs III/IV)	(1/2 vs 3)		(P vs N)	(P vs N)	(P vs N)	mRNA
ERα standard	correlation	−0222*	−0.317**	N.S	N.S	N.S	N.S	−0.214*
	coefficient							
	p (2-tailed)	0.033	0.001	N.S	N.S	N.S	N.S	0.022
	N	92	116	116	109	86	50	115
ERα∆7	correlation	−0.223*	−0.203*	N.S	−0.206*	N.S	−0.332*	−0.168
	coefficient							
	p (2-tailed)	0.033	0.029	N.S	0.032	N.S	0.019	0.073
	N	92	116	116	109	86	50	116
ERα∆7/standard	correlation	−0.251*	−0.227*	N.S	−0.232*	N.S	−0.407**	−0.198**
	coefficient							
	p (2-tailed)	0.016	0.014	N.S	0.015	N.S	0.003	0.0034
	N	92	116	116	109	86	50	116

T = Primary tumor; LN = lymph node metastasis; M = distant organ metastasis; L = lymphangiosis; P = positive; N = negative; p = p value; N.S = Not significant; * = significant at the 0.05 level (2-tailed); ** = significant at the 0.001 level.

**Table 3:** ***ERaD7*** **mRNA level in correlation to different parameters (Kruskal-Wallis Test)**

		FIGO	Grade	LN	L	hTra2β1
		(I/II vs III/IV)	(1/2 vs 3)	(P vs N)	(P vs N)	
ERα standard	mean rank	50.97 vs 38.87	63.74 vs 37.33	56.59 vs 44.21	29.25 vs 22.04	62.31 vs53.61
	p (2-tailed)	0.034	0.001	0.168	0.075	0.159
ERα∆7	mean rank	50.90 vs 39.00	61.77 vs 45.26	57.42 vs 38.57	30.23 vs 21.13	62.62 vs 53.30
	p (2-tailed)	0.034	0.030	0.032	0.020	0.123
ERα∆7/standard	mean rank	51.48 vs 38.00	62.17 vs 43.67	57.73 vs 36.46	31.31 vs 20.13	72.52 vs 49.00
	p (2-tailed)	0.016	0.015	0.016	0.004	0.000

### The ratio of ERaD7 to ERa standard is inversely related to HTRA2-BETA1 expression

We chose real-time PCR quantification of ERaD7 isoform since this methodical approach results in more accurate data on mRNA quantity. Real-time qPCR runs as a robust and reliable standard procedure in our lab and all randomly applied re-checks of qPCR products via classic gel electrophoresis accounted for the desired amplicons. Since our real-time PCR sense primer for *ERaD7* detection was designed to be complementary to the conjunction of exon6 and 8, the calculation of *ERaD7* mRNA level might be influenced by exon6 skipping, even though there were only 8 samples positive for *ERaD6*. To overcome this problem, we also calculated the expression ratio of *ERaD7* in total *ERa* transcript amount (ratio = *ERaD7* mRNA level/*ERa* standard mRNA level) and performed additional analyses. Significant differences in the ratio between moderately and poorly differentiated, FIGO stage III/IV as well as lymph node positive tumors (p = 0.015, p = 0.016. p = 0.016, respectively, Kruskal-Wallis Test, Table 3) could be detected. Like the *ERaD7* mRNA level, its relative expression ratio in total *ERa* was also found to be associated with FIGO stage (R2 = 2.311, p = 0.006, Additional file 1: Table S3). Furthermore, we were able to detect an inverse correlation of *ERaD7*/*ERa* standard ratio with *HTRA2-BETA1* mRNA levels (correlation coefficient = −0.198, p = 0.034, Table 2).

### Higher ERaD7 mRNA levels are associated with improved survival

Expression of *ERa* and its isoform *ERaD7* were also analyzed in regards to patient outcome. Besides the earlier mentioned categorization in groups 0–2 an additional *ERaD7* expression ratio was defined with two groups: *ERaD7*/*ERa* standard mRNA ratio >0.5 and *ERaD7*/*ERa* standard mRNA ratio ≤0.5.

**Figure 3:**
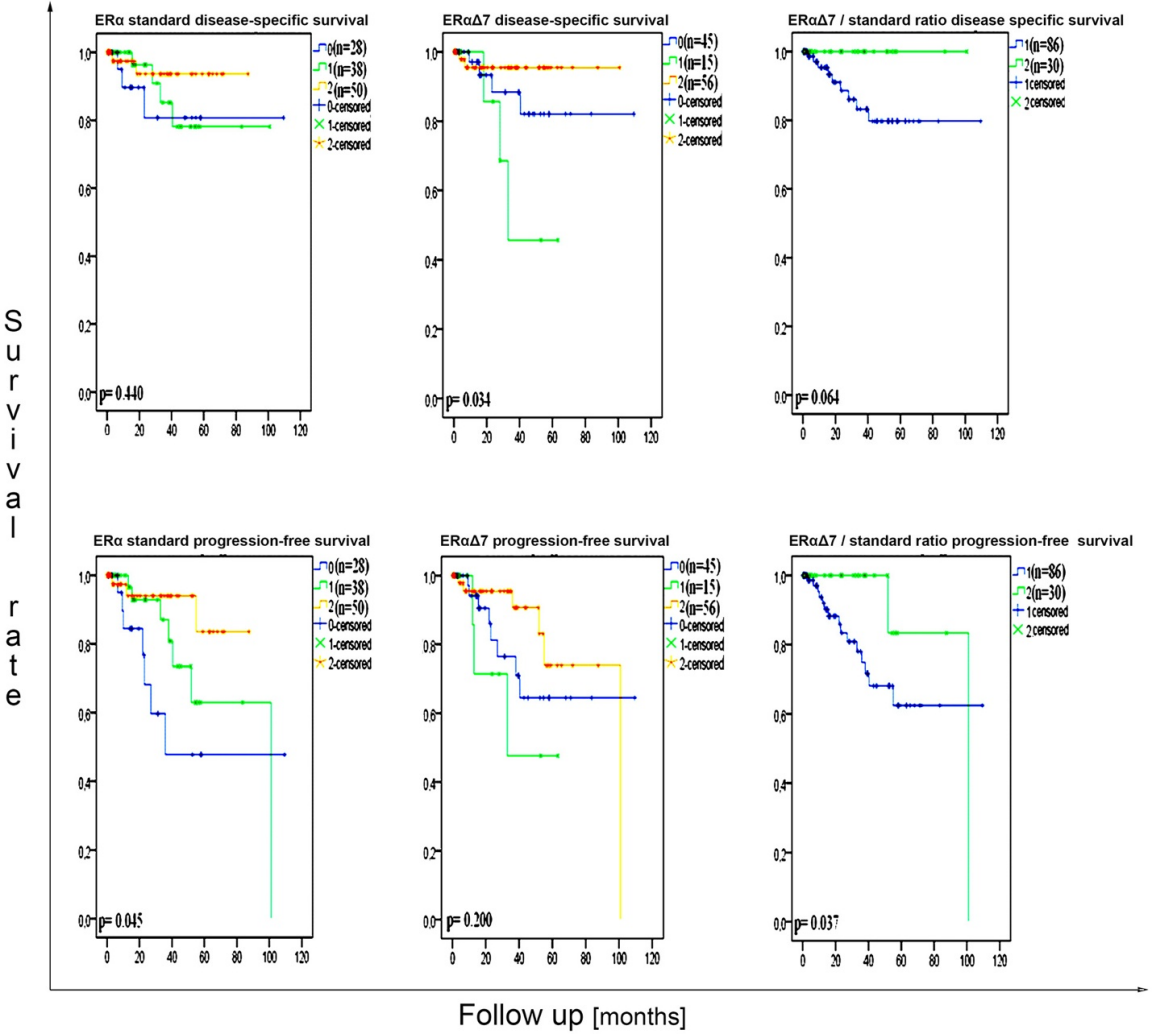
**Kaplan-Meier survival curves for disease-specific survival and progression-free survival in regard to*ERa*standard and*ERaD7*expression (group 0: no ERa standard mRNA detectable; group 1: ERa standard mRNA amount ≤0.81; group 2: ERa standard mRNA amount >0.81, due to the mean mRNA level within ERa standard positive samples being 0.81.** ERaD7 mRNA levels were defined as group 0: no ERaD7 mRNA detectable; group 1: ERaD7 mRNA amount ≤0.80; group 2: ERaD7 mRNA amount >0.80, due to the mean ERaD7 mRNA level within ERaD7 positive ones being 0.80.) Log rank test.

Univariate survival analyses suggested that patients with higher *ERa* expression had a better progression-free survival (p = 0.045, Figure 3). Patients with high *ERaD7* mRNA levels (group 2) displayed a better cumulative survival rate in comparison to level 1 and level 0, respectively. This difference correlated with improved disease-specific survival (p = 0.034, Figure 3). In line with these findings higher *ERaD7*/*ERa* standard ratio were correlated to an improved progression-free survival rate (p = 0.037, Figure 3).

When performing Cox regression test, data were adjusted for *ERa* and *ERaD7* mRNA level groups, FIGO stage (I/II versus III/IV), tumor differentiation grade (G1/G2 versus G3), lymph node metastasis and distant organ metastasis, respectively. The latter two factors were entered as categorical variables defined as negative, positive, and unknown status. As expected, differentiation grade was identified as an independent prognosticator for disease-specific survival, but *ERa* standard mRNA expression was also identified as an indicator for progression-free survival (Additional file 1: Table S3), respectively.

## Discussion

ERa regulates gene expression either by binding to estrogen response elements (ERE) or through ERE-independent signaling (such as interactions with other transcription factors like AP-1, SP1, NF-KB) [20-24]. Those downstream effects from aberrant ERa regulation provoke changes in cellular function toward carcinogenesis. ERa as a prognosticator for EC has been studied for its potential influence on EC carcinogenesis. Horvath and colleagues reported a decrease of wild type *ERa* and an increase of *ERaD7* isoform in estradiol treated EC tissue correlated with an estradiol-resistant growth phenotype with no growth rate change in comparison to controls [25]. These findings together with others suggested a competitive effect of ERaD7 on its wild type in determination of cellular hormone sensitivity [10,13]. Our study revealed an ERaD7 induction in type 1 EC and a correlation of its expression level to the FIGO stage. Higher *ERaD7* mRNA levels were statistically significant correlated to an improved outcome with a better disease-specific survival as demonstrated by Kaplan-Meier survival curves (p = 0.034, Log-rank test, Table 3). The univariate survival analyses demonstrated a significant improved progression-free survival, defined as incidence of local or distant recurrence, for total ERa expression, which was already published in the literature. For ERaD7 a significant improved disease-specific survival, excluding all non-endometrial cancer related deaths, was also seen. In line with these observations the ratio of ERaD7/total ERa transformed into an improved progression-free survival (p = 0.037) in this EC subtype.

ERaD7 origins from an out-of-frame exon deletion that disrupts the ligand binding domain. Therefore supposedly functions as a dominant negative repressor of ERa transactivating properties [8]. Jazaeri et al. mention that ERa variants, e.g. ERaD7, may account for growth advantages in variant expressing cells under selective pressure caused by estrogens or anti-estrogens [8]. Furthermore they point out, that according to the heterodimer activity of ERa standard (wild-type) and variants, even small quantities of alternatively spliced isoforms can have a major effect on cell physiology [8]. We hypothesize, that the correlation of elevated ERaD7 expression and improved outcome in type 1 EC is based on the diminished cellular estrogen sensitivity. Malfunctioning estrogen receptor a-dependent transcription and associated tumor progression signaling pathways could account for the decrease of malignant behavior of ERaD7 expressing endometrial tumors. Furthermore, the pharmacological effect of anti-estrogens, e.g. tamoxifen, might be also reduced by ERaD7-mediated ERa resistance in regard to transcriptional activation of target genes. Functional studies demonstrated increased cellular levels of ERaD7 in response to both estrogen and tamoxifen exposure [25]. Interestingly, long-term exposure to either unopposed estrogen, e.g. hormone replacement therapies or tamoxifen treatment are major risk factors for EC [26].

So far the underlying mechanisms of regulation of *ERa* exon7 expression were not understood. Gotteland and colleagues described different *ERa* mRNA isoforms in physiological and malignant breast tissues, suggesting this phenomenon could be caused by alternative splicing, independent from cell transformation [27]. The analyses of the *ERa* exon7 sequence strongly supported the hypothesis of alternative splicing regulation (Figure 1A, B). Both, its 5’ polypyrimidine tract and 3’ intron sequence contain HNRNP I binding motifs [28-30]. It is known that HNRNP I represses exon splicing by looping out exons between its binding motifs, which has been found in various kinds of tissues (reviewed in [31-34]). This might explain why *ERaD7* is the most frequently detectable isoform of *ERa*. It is well known that HTRA2-BETA1 preferentially promotes splicing of exons with GAA-rich domains in a concentration dependent manner [35,36]. The *ERa* exon7 sequence expresses two potential HTRA2-BETA1 binding motifs which could explain why HTRA2-BETA1 is promoting exon7 splicing on both, the endogenous as well as the exogenous level. HNRNPG is a known antagonizing factor of HTRA2-BETA1 activity in mRNA processing [37]. In our *in vitro* analyses, the expected antagonizing effects of HNRNPG on HTRA2-BETA1 became evident by the specific induction of *ERa* exon7 skipping (Figure 2B, D, G). Since exon7 contains the preferential HNRNP G binding sites AAGU and CC(A/C) [37,38] we hypothesize in accordance to other groups [37], that both splicing factors HTRA2-BETA1 and HNRNP G exhibit their antagonistic effects on *ERa* exon7 splicing by a concentration dependent competition (Figure 1C).

In previous studies, we analyzed ERa alternative splicing pattern in EC in regard to skipped exons or exon cassettes by use of combinatory primer pairs for PCR. Our prior analyses did not identify exon7/exon 8 skipping in EC samples, in detail: no EC cell line or EC tissue specimen (>20 specimen tested) exhibited this splicing possibility (data not shown).

Carcinogenesis is characterized by complex alterations in a magnitude of cellular mechanisms. Aberrant alternative splicing has a high impact on cellular processes that lead to cancer or promote cancer progression, including resistance to apoptosis and promotion of invasion, metastasis and angiogenesis, respectively [15]. Our previous study demonstrated that HNRNP G and HTRA2-BETA1 trigger opposite effects on EC prognosis: a simultaneous higher level of HTRA2-BETA1 protein nuclear expression as well as mRNA is correlated to poor disease-specific as well as progression-free survival. On the contrary, high expression levels of nuclear HNRNP G protein and mRNA are associated with an improved clinical outcome in the same patient cohort. In our present study, we detected an inverse correlation between *ERaD7* expression ratio and *HTRA2-BETA1* mRNA level. Furthermore, our *in vitro* experiments demonstrated that HTRA2-BETA1 works as a splicing enhancer for *ERa* exon7, while HNRNP G acts as an opponent of HTRA2-BETA1 by antagonizing the HTRA2-BETA1 effect on *ERa* exon7 inclusion.

These functional data are in line with our observation regarding the correlation of *ERaD7* expression and the clinicopathological features as well as outcome data of patients with type 1 EC.

## Conclusions

The present study strongly supports our recently published hypothesis, that increased *HNRNPG* levels are associated with improved clinical outcome. This is due to the fact, that we were able to identify this nuclear protein as a specific regulator towards high levels of *ERaD7* expression. However, the best proof for this theory is given by the fact that increased expression of *ERaD7* was also characterized as a prognosticator towards an improved clinical outcome. The important biological role of ERa in estrogen-dependent EC carcinogenesis is further supported by our study.

Taking all evidence into account, we hypothesize that expression pattern of splicing factors have profound effects on cancer cell biology. Our present study provides a new evidence for the pivotal impact of aberrations in alternative splicing pattern in carcinogenesis.

## Additional file

Additional file 1: **Table S1.** Primers for real time and conventional PCR. **Table S2.** Plasmid transfection quantities. **Table S3.** Correlation of ERaD7 mRNA level with FIGO stage (Multivariate general linear regression test). **Table S4.** p value of ERa exon7 skipping/inclusion and HNRNP G/HTRA2-BETA1 mRNA ratio difference in differently treated cells.

## Abbreviations

cDNA: Complementary deoxyribonucleic acid
EC: Endometrial cancer
ERa: Estrogen receptor alpha
ERaD3: Estrogen receptor alpha delta 3, splice variant
ERaD4: Estrogen receptor alpha delta 4, splice variant
ERaD5: Estrogen receptor alpha delta 5, splice variant
ERaD7: Estrogen receptor alpha delta 7, splice variant
ERE: Estrogen response elements
ESR1: Estrogen receptor 1, estrogen receptor alpha
FIGO: International Federation of Obstetrics and Gynecology
HKG: Housekeeping gene
hnRNP G: Heterogeneous ribonucleoprotein particle G
hnRNP I: Heterogeneous ribonucleoprotein particle I
hTra2-beta1: Human Transformer-2 sex-determining protein – beta1
INS: Insulin
pCMV: Plasmid containing Cytomegalovirus sequence
real-time quantitative PCR: Real-time quantitative polymerase chain reaction
RT-PCR: Reverse transcription - polymerase chain reaction
