# Supplementary material for: HNRNP G and HTRA2-BETA1 regulate estrogen receptor alpha expression with potential impact on endometrial cancer
Source: BMC Cancer. 2015 Feb 27;15:86. doi: 10.1186/s12885-015-1088-1 (PMC4355463; doi:10.1186/s12885-015-1088-1)
Supplement: Additional file 1: — Table S1. Primers for real time and conventional PCR. Table S2. Plasmid transfection quantities. Table S3. Correlation of ERaD7 mRNA level with FIGO stage (Multivariate general linear regression test). Table S4. p value of ERa exon7 skipping/inclusion and HNRNP G/HTRA2-BETA1 mRNA ratio difference in differently treated cells. [file 12885_2015_1088_MOESM1_ESM.docx]

Additional file

**Table S1:** Primers for real time and conventional PCR

**Table S2:** Plasmid transfection quantities

**Table S3:** Correlation of ERaD7 mRNA level with FIGO stage (Multivariate general linear regression test)

**Table S4:** p value of ERa exon7 skipping / inclusion and HNRNP G / HTRA2-BETA1 mRNA ratio difference in differently treated cells.
